# Interdisciplinary Approach to Tool-Handle Design Based on Medical Imaging

**DOI:** 10.1155/2013/159159

**Published:** 2013-09-19

**Authors:** G. Harih, A. Čretnik

**Affiliations:** ^1^Laboratory for Intelligent CAD Systems, Faculty of Mechanical Engineering, University of Maribor, Smetanova Ulica 17, 2000 Maribor, Slovenia; ^2^Department of Traumatology, University Medical Centre Maribor, Ljubljanska Ulica 5, 2000 Maribor, Slovenia; ^3^Department of Surgery, Faculty of Medicine, University of Maribor, Slomškov trg 15, 2000 Maribor, Slovenia

## Abstract

Products are becoming increasingly complex; therefore, designers are faced with a challenging task to incorporate new functionality, higher performance, and optimal shape design. Traditional user-centered design techniques such as designing with anthropometric data do not incorporate enough subject data to design products with optimal shape for best fit to the target population. To overcome these limitations, we present an interdisciplinary approach with medical imaging. The use of this approach is being presented on the development of an optimal sized and shaped tool handle where the hand is imaged using magnetic resonance imaging machine. The obtained images of the hand are reconstructed and imported into computer-aided design software, where optimal shape of the handle is obtained with Boolean operations. Methods can be used to develop fully customized products with optimal shape to provide best fit to the target population. This increases subjective comfort rating, performance and can prevent acute and cumulative trauma disorders. Provided methods are especially suited for products where high stresses and exceptional performance is expected (high performance tools, professional sports, and military equipment, etc.). With the use of these interdisciplinary methods, the value of the product is increased, which also increases the competitiveness of the product on the market.

## 1. Introduction

Design is a very complex task even for an experienced designer. Technological innovation pushes the creativity to new limits and therefore designers are forced to design new products with new functionality within ever shorter time to market deadlines [1]. Products are usually mass produced in order to keep the production costs at lowest level and are therefore designed to suit a wide population. However, there has been an increased market demand of customized products, which incorporate whole product customization or customized parts for a target population [2]. Major part of companies or institutions who utilize customization can be attributed to medical applications (medical prosthesis, implants, splints, etc.). Recent extensive development in various technologies such as medical imaging, 3D scanners, and rapid prototyping has impacted also customization within luxury products and in products where high stresses and exceptional performance are expected such as high-performance tools and gear, professional sports equipment, and military equipment. 

In order to produce a customized part for a target population, the designer has an even more complex design process to overcome. The designer has to consider the product-human interaction in order to develop products with high rate of efficiency and comfort [3]. In a human-product interaction, the designer has three constraints, which have to be considered to design an efficient product. Design attributes of the product define the task and product constraints; the cognitive and biomechanical constraints are defined with the user. If there is a viable human-product interaction possible, all three constraints must overlap to some extent. Somewhere inside the intersected area is the optimal human-product interaction for the target population. To find the optimum, the designer has to set his objective function and perform optimization. Task and product constraints can be altered with different design attributes; therefore, a designer has to have knowledge about the target population's biomechanical and also cognitive constraints in order to adapt the product design for the optimal human-product interaction which consists of expected product functionality, performance, and also safety. 

Ergonomic principles should be included in the phase of industrial/mechanical product design before the engineers tackle the problem, because the main function of the product and the form of the product are usually strongly connected [3, 4]. Since product ergonomics is an interdisciplinary science, the designer has to possess wide range of knowledge and also experience to allow a holistic design approach to reach the expected human-product performance and safety [5]. Modern CAE and CAD software allow the designer to evaluate the new product virtually. In the field of workplace ergonomics, many software solutions exist, although there is still a lack of dedicated ergonomics software in the field of product ergonomics and design, which would make evaluation and analysis of the proposed design at the virtual stage possible. 

Traditional user-centered design techniques such as designing with recommendations, designing based on anthropometric data, and derived mathematical models do not incorporate enough subject data to design a customized product with best fit to a specific target population. To overcome limitations of traditional design, there has been an increase in use of interdisciplinary medical imaging approach to reverse engineer 3D computer-aided design (CAD) models of human anatomical parts to incorporate them into the design process in CAD software or to utilize finite element analyses (FEA). For example, 3D models of human foot based on medical imaging are increasingly being used in the design process of footwear. They are used to utilize CAD and FEA in order to optimize the performance and comfort rate of the users [6]. There has been also an extended use of medical imaging in generating 3D digital human models, which can be utilized for user-centered design, although they are usually used for workplace ergonomics and cannot be used in customized product design. 

A huge part of work is still manual work using hand tools. One of the key functions of the hand is the interaction with the physical environment, where the most important is the prehensile hand grasp with a physical object. In this way, it is effectively used as a tool for work, as well as the interface to use various powered and nonpowered hand tools and equipment. Due to the nature of certain complex tasks, where the grasp of certain tool is necessary, the loads on the hand are high as well (vice-versa), which can lead to discomfort, pain, and acute and cumulative trauma disorders (ATD and CTD, resp.). The most common disorders include nerve and tendon disorders (carpal tunnel syndrome, picondylitis, tendinopathies such as peritendinitis, tenosynovitis…), hand-arm vibration syndrome, and some pressure-related (blisters, ulcers), ischemic, or other problems [7–11]. This presents high costs for the company with sick leave of the workers and high costs with diagnostics and treatment [12, 13]. To prevent these ATD and CTD, the product development engineer has to consider the recommendations in designing ergonomic handles of hand tools.

Most authors have provided recommendations and mathematical models for cylindrical handle design. Different authors used different criteria to determine optimal cylindrical handle: subjective comfort rating [14, 15], finger force measurement [16, 17], muscle force minimization [18], and hand anthropometrics [14, 19–24]. Few studies also used two or more criteria: finger force measurement and muscle activity [19, 22, 25], subjective comfort rating, finger force measurement, and electromyographic efficiency of muscle activity [26]. However, none of the authors considered the shape of the hand in the design process, which could additionally improve the ergonomics of the handle. It has been shown that handles should vary in size between hand and finger size, since maximum possible exerted finger force is diameter dependent [26]. It has been also shown that objects that follow the shape of the hand result in much lower local contact pressures of the soft tissue, which can prevent discomfort and several disorders [27]. Authors suggested that further research of this topic should consider the shape of the hand in the optimal power grasp posture since it could improve the ergonomics of the tool handle [23]. 

The shape of the handle cannot be determined using traditional methods with recommendations, anthropometric measurements, or simple mathematical models. Therefore we present methods based on medical imaging to develop a tool handle with optimal size and shape for a target population. Methods allow high consideration rate of biomechanical constraints and try to reach the optimal human-product interaction for increased performance and comfort. Described methods are especially useful for product development where the use of traditional user-centered design methods is not possible because of the nature of the human body.

## 2. Material and Methods 

### 2.1. Three-Dimensional Data Acquisition 

Three-dimensional data acquisition in reverse engineering is usually performed with methods such as coordinate measuring systems and increasingly often with new methods such as laser and optical scanners. Because of nature of the human body, the most used method in tissue three-dimensional data acquisition is magnetic resonance imaging (MRI) and computed tomography (CT) [28]. CT still provides images with higher spatial resolution; however, MRI provides images with better contrast resolution. MRI is according to health risk way superior to CT, since it does not expose the subject to ionizing radiation. 

**Figure 1:**
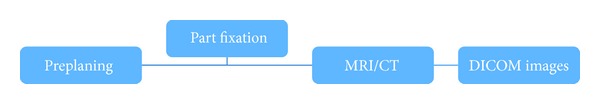
Three-dimensional data acquisition for customized product design.

Because of the long imaging times, the imaged anatomical part should be fixated properly. If movement occurs during the imaging, images may be distorted. This can result in images that do not reflect the true anatomical structure of the subject. It also makes the segmentation and 3D reconstruction difficult or impossible. For applications, where a specific posture of the imaging anatomical part has to be maintained, a mould should be considered for the fixation. Orthotic moulds are most appropriate, since they have good ability to mould to anatomical contours. Modern CT/MRI machines usually provide images in the DICOM file format (Digital Imaging and Communications in Medicine) where distance between each slice can be set to 1 mm or even less. Lower slice distance theoretically provides more anatomical details and therefore provides better starting point for segmentation and 3D reconstruction, but it also extends the time of imaging. If the imaging time is long and subject has to hold an uncomfortable position of the imaged anatomical part, muscle twitching can occur, which affects the accuracy of the images. Therefore, it is crucial to find a compromise between the slice distance and imaging time. Based on the literature and experiments, the optimal time differs from anatomical part, muscle type, posture, and subject, but in most cases it should not exceed ten minutes. The process of three-dimensional data acquisition is schematically displayed in Figure 1.

**Figure 2:**
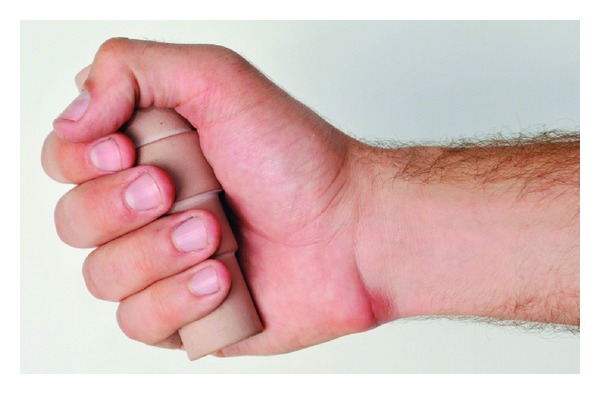
Testing the optimal cylindrical handle with variable diameters.

As shown in the introduction, the shape of the tool-handle cannot be determined using traditional methods with recommendations, anthropometric measurements, or simple mathematical models. Therefore, we utilized methods based on medical imaging to develop a tool handle with optimal shape to increase the performance, comfort, and the stability in the hand. We considered power grasp, which is mostly suited for various powered and also nonpowered hand tools [29]. Firstly, anthropometric measurements were performed to determine optimal diameters for each finger to maximize grip force and comfort [23, 26, 30]. Afterwards, an optimal cylindrical prehandle with variable diameters was manufactured (Figure 2). 

**Figure 3:**
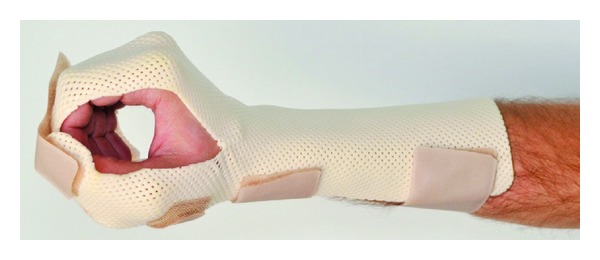
Top view of the outer hand mould attached to the hand.

To obtain the shape of the hand in its optimal power grasp posture with undeformed soft tissue, an outer hand mould out of orthotic material was manufactured on the dorsal side of the hand while softly holding the optimal prehandle. The mould maintains the diameters and the shape of the hand in its optimal power grasp posture (Figure 3).

The MRI was performed at the Department of Radiology, University Clinical Centre Maribor using the GE medical systems SignaHDxt 3.0T MRI machine. The optimal imaging time was found to be lower than ten minutes to avoid muscle twitching; therefore, slice thickness of 2 mm was set. This allowed precise segmentation and also proved to deliver accurate enough results. The subjects were asked to hold the hand in open position fully touching the mould during the imaging to maintain the proper diameters and shape of an optimal power grasp. Image area was 512 × 512 × 121 pixels. Scanned images were provided in DICOM format.

### 2.2. 3D Reconstruction

**Figure 4:**
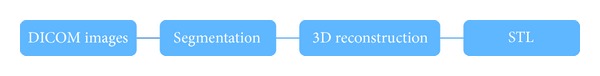
Process of 3D reconstruction on the obtained images.

After the data acquisition based on CT or MRI, the images have to be 3D reconstructed in order to obtain a CAD model of the imaged anatomical part. 3D reconstruction for CAD modeling is usually performed with segmentation, which is a process where digital images are being partitioned into multiple segments to determine the areas of specific anatomical part [28]. Each segmented image is then stacked together with the imaging slice distance. The stack of segmented images is then a reference for solid reconstruction. Many segmentation algorithms have been developed in order to improve the speed and accuracy of the segmentation process; however, we only discuss the relevant segmentation techniques to CAD or FEA model development. The simplest segmentation is with thresholding levels to segment a specific anatomical region. If no anatomical differentiation is needed on the imaged anatomical part, segmentation based on thresholding is the fastest and simplest method to extract the shape of the imaged part and develop a CAD model. If differentiation of anatomical parts is needed to construct a biomechanical CAD model to perform kinematic analyses or FEA, segmentation techniques such as region growing or similar should be used. Because of the nature of imaging techniques, noise and other image errors are usually present; therefore, the designer has to utilize noise and other image-cleaning tools. After the segmentation and 3D reconstruction, the obtained 3D representation has to be exported in a file format that is supported by CAD software of choice. Most of the modern medical imaging software allows export of STL (stereolithography) file format of the 3D reconstructed model. Workflow of 3D reconstruction process can be seen in Figure 4.

Rapid development of medical imaging has influenced also the development of various professional medical imaging and biomedical research and development software. Most software packages provide comprehensive tools for 2D image manipulation, segmentation, measurement, 3D reconstruction, and so forth. Majority of the software is commercial, such as Mimics by Materialise, Amira by Visage Imaging, and MedCAD, but there are also some free open source software packages such as 3D Slicer [31–35]. 

For the segmentation and 3D reconstruction of the obtained DICOM images, Amira 5.3.3. (Visage Imaging) was used. Segmentation was performed using threshold technique, since only surface of the hand is relevant. LabelVoxel module with threshold value of 200 was used for the segmentation, which has been proved to be the best value to obtain best segmentation. Small inclusions and segmentation errors were corrected with “remove islands” and “fill holes” commands. 

**Figure 5:**
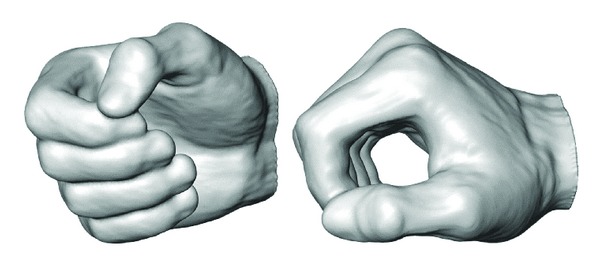
Obtained 3D hand in Amira in optimal power grasp posture.

3D reconstruction showed that the model is anatomically accurate and the produced outer surface is very detailed. In order to get smoother surface, a “resample” module was added to the segmentation and 3D reconstruction process. Additionally, the hand was cropped to the wrist, since only specimen's hand is relevant to the study. Open source imaging software 3D Slicer was used to verify the resulting 3D reconstruction [32]. The result is a smooth 3D representation of the specimen's hand in an optimal power grasp posture (Figure 5). 3D reconstructed hand was then exported in STL file format.

### 2.3. Reverse Engineering and CAD, FEA

The obtained STL model does not include any geometric topological relations. Therefore, no feature-based CAD solid modelling techniques and no FEA are possible on the obtained STL model, which require vector-based modelling environment [28]. The obtained 3D reconstructed model of the imaged anatomical part has to be reverse engineered into a nonuniform rational B-Spline (NURBS) function. Some of the medical imaging software already includes reverse engineering modules and some solid modelling techniques, although the modelling is usually attributed to implant modelling, surgical planning, and so forth and is not suited for the product design modelling. Therefore, in product design based on medical imaging, it is necessary to use CAD software, such as standalone reverse engineering software (Geomagic by Raindrop Inc., etc.) or CAD software packages with reverse engineering modules or plug-ins (Solidworks with Rapidform plugin, Catia). 

The point data from the STL file has to be triangulated in the reverse engineering software to form a freeform NURBS surface model. The result is usually a surface model which consists of surface patches. The surface model is then converted into a solid model which enables classic solid CAD modeling. Since most of the human anatomical parts are organic shapes, feature recognition on the CAD model is not appropriate; therefore, a solid model is sufficient. 

**Figure 6:**
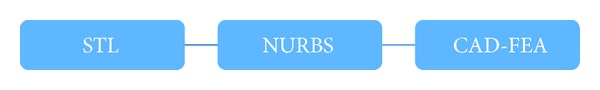
Needed processes for CAD model extraction from medical images.

In the CAD process of designing the customized product with best fit, a rough solid model which will be in interaction with the subject is then modeled and positioned over the obtained CAD model of the anatomical part. Boolean operations (Add, Remove, and Intersect) are then being used to define the intersecting volume and thereby get the shape of the product with perfect fit. After the shape has been obtained, the designer can use usual CAD tools to improve the design of the customized product based on his skills and experiences. The outline of processes to utilize CAD-FEA with medical imaging can be seen in Figure 6.

**Figure 7:**
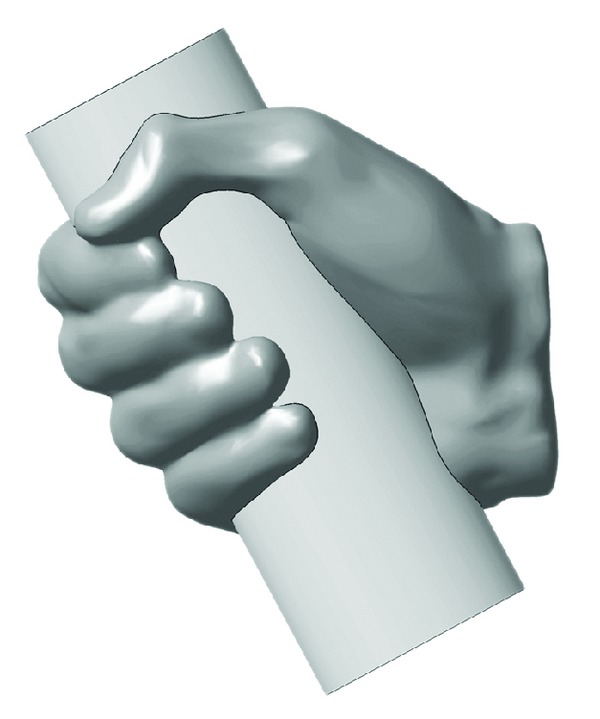
3D hand and elliptical cylinder in overlapping position.

In our case, the obtained STL file was imported into commercial CAD software CATIA V5R20 where the reverse engineering module Quick Surface Reconstruction was used to obtain NURBS model. An elliptical handle was modeled with the size and position according to the empty ventral opening of the obtained 3D hand (Figure 7). The handle was then obtained with Boolean operation, which removed the cylinder model volume, which was in overlap with the hand model volume.

**Figure 8:**
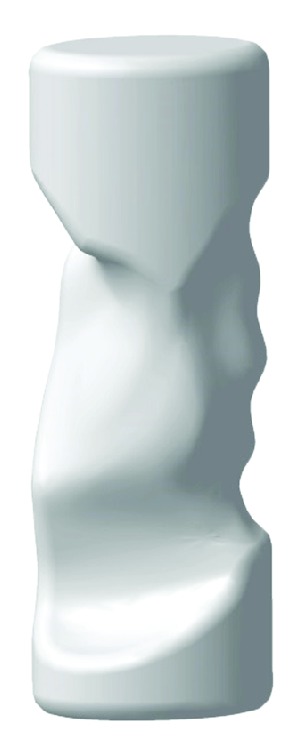
Custom shaped handle with higher amount of smoothing and rounded edges.

The result is a handle with perfect fit to the target subject. Although resample module was added in the 3D reconstruction process, the resulting handle is very detailed, which is the result of the surface details of the scanned hand. It is reasonable to assume that small anatomical details do not contribute to the increase of subjective comfort rate and increase of contact area; therefore, from manufacturing and aesthetic considerations, additional smoothing was applied to the 3D reconstructed hand. The result is smoother and aesthetically more appealing handle (Figure 8). 

**Figure 9:**
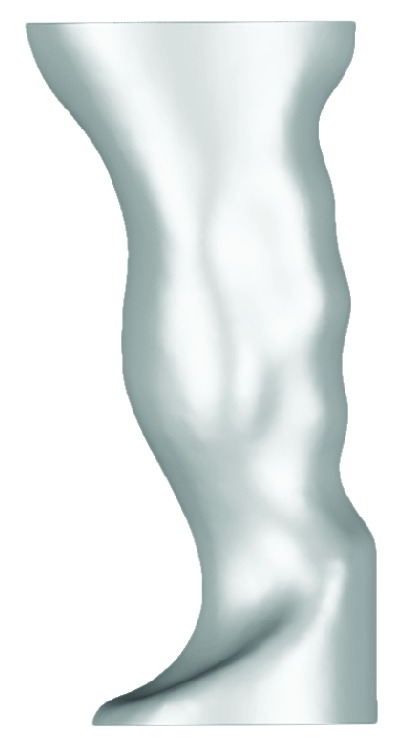
Symmetrical tool handle.

The obtained tool handle was edited in order to obtain a symmetrical handle, which allows usage with both hands. Therefore, a cutting/mirroring plane was modeled. The position of the cutting plane was determined to maintain the optimal diameters of the resulting symmetrical tool handle. Afterwards the plane was used to cut the thumb side of the handle. The same plane was used to apply a mirroring function of the remaining handle shape to obtain a symmetrical handle. Low smoothing was applied locally to correct small topological irregularities (Figure 9). 

Additionally, a generalized tool handle was developed based on local topological smoothing, which allows the usage by broader population. The generalized handle is more likely to produce higher local contact pressures, since the shape of the handle does not exactly follow the shape of the hand of the target user due to topological smoothing.

### 2.4. Manufacturing and User Testing

**Figure 10:**
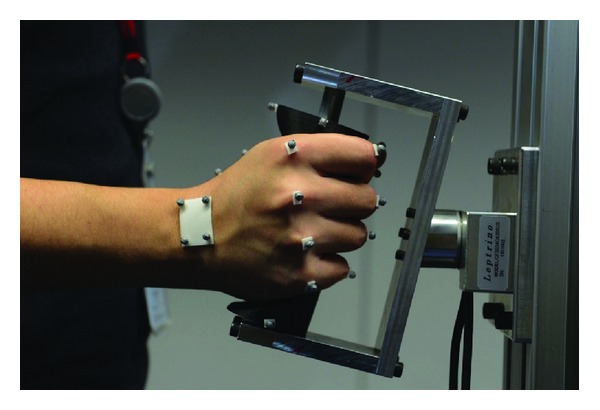
User testing the obtained tool handle.

In order to compare the obtained tool handle based on medical imaging with a traditional designed cylindrical handle, ten handles were manufactured using 3D printing technology. Each corresponding handle was given to test subjects who performed a generalized gripping task consisting of one minute push and afterwards pull force of 50 N, then torque left 1 Nm and torque up for 2 Nm and also maximum voluntary contraction for ten seconds (Figure 10). Motion data was also recorded during the gripping task using motion capture system for the comparison. However, the data and results of motion capture are out of the scope of this paper. The test subjects were afterwards given a subjective comfort rating questionnaire to assess the comfort rating of both handles [36]. Additionally the generalized handle has been manufactured and tested on subjective comfort rating.

## 3. Results and Discussion

The obtained handle was virtually evaluated inside the CAD software. The evaluation process included the measurement and verification of the optimal diameters for maximizing the grip force and comfort and measurement of the contact area. It has been shown that the diameters were withheld by the outer hand mould since there was low deviation from the optimal diameter for each finger. Therefore, also maximum grip force can be exerted, which increases the user performance while using the newly developed tool handle. Subjective comfort rating is also maximized, since there is only small difference in calculated optimal diameters for each finger. The 3D reconstructed hand therefore represents the optimal power grasp posture of the subject, which was the basis for obtaining an optimal shaped tool handle.

The contact area while gripping the tool handle was measured virtually inside the CAD software and was compared between both handles. The mean contact area of the anatomical handle was on average 25% higher than the cylindrical handle. The increase of the contact area found in calculations could be expected, since the optimal shaped handle follows the anatomical shape of the hand in its optimal power grasp posture and therefore the contact area is maximized. It is clear that contact area maximization is not possible with cylindrical handles when considering optimal diameter to maximize grip force exertion and comfort rating. It could be therefore concluded that contact area maximization is only possible when considering the anatomical shape of the hand in its optimal power grasp posture. Thereby greater contact area can be obtained lowering overall and local contact pressure. It is also likely that the higher comfort rating can be attributed to the higher contact area and anatomical shape of the handle. This could also probably lead to prevention of ATD and CTD (like pressure ulcers, overuse injuries, and vibration diseases) and provide greater comfort rate than a cylindrical handle.

Contact area maximization seems to be crucial to lower the local and overall contact pressure and thus could bring to diminish ATD and CTD as described in Section 1. Contact area maximization and therefore contact pressure minimization should be highly striven as skin and subcutaneous tissue have nonlinear viscoelastic properties and the stresses increase exponentially with the increase of strains. Increased stability of the obtained optimal shaped handle of the handle could be also expected since most of the external forces and torques are transferred with the shape of the handle and much less with friction as with the optimal cylindrical handle. Therefore, lower normal grip force could be exerted in comparison to the cylindrical handle with less evident slippage and rotation.

Test subjects provided a subjective opinion regarding comfort rating of both handles. A dependent samples *t*-test was conducted to examine whether there was a significant difference between both handles. 

It has been shown that there is significant difference in the comfort regarding Fits the hands and Offers nice grip feeling. This can be explained by the anatomical shape of the handle, because the anatomical handle considers the optimal power-grasp posture, with optimal diameters being achieved for each finger, which assures the maximum voluntary contraction of fingers. This was impossible with the cylindrical handle, since it took only one finger's optimal diameter determination into account. Statistical significant difference can be also observed in terms of stability. The majority of the forces and moments are transferred over to the anatomical handle shape and much less with the friction between the handle material and skin; therefore, the subjects rated the anatomical handle as more stable. It can be also assumed that the normal gripping force of a cylindrical handle would be therefore higher to prevent slippage of the handle. High local and overall contact pressures occur from highly exerted normal forces that can cause discomfort and also acute disorders and CTD (i.e., blisters, inflamed skin, cramped muscles, etc.). In terms of overall comfort, the subjects rated the obtained anatomical handle as more comfortable than cylindrical. 

It has been shown that the generalized handle provides higher comfort rating than cylindrical handles but is less comfortable than the customized tool handle. It has been also shown that increased comfort rating of the generalized tool handle becomes less evident with subjects who have greater deviation to the optimal diameters of the generalized tool-handle. 

In user-centered design as described in the present study, medical imaging was the only viable option for the acquisition of accurate 3D hand surface with undeformed soft tissue to develop optimal sized and shaped tool handle. Developed methods take also into account the shape of the hand in its optimal power grasp posture and therefore provide best fit for the target population's hand, which is novelty in the field of ergonomics. All current studies are concentrated on optimal diameters but lack the shape determination, which has been shown to increase the user comfort, stability, and performance and is therefore more likely to prevent ATD and CTD.

Methodology described in this study is the first seen interdisciplinary approach to tool-handle design, which allows determination of the optimal handle shape. It has been shown that any institution or company with access to biomedical department with an MRI or CT machine can utilize the described methodology to determine and manufacture optimal sized and shaped products for target population. Quality and reliability of this technique can be used for the innovative design process to develop customized products which allow high consideration rate of biomechanical constraints and therefore provide best fit to the target population. The medical approach is especially suited for integrated CAE/CAD design process and FEA utilization since product design is mostly done inside computer software. MRI and CT machines are usually used for medical diagnostics, orthotic materials for orthoses, but the usability of these technologies and medical materials can be successfully extended by interdisciplinary studies and production methods as described in this study. Thereby, the value of the machines and materials is also increased, which affects the amortization time of these machines. The proposed methods fulfil the requirements of the market for product customization and shape optimization, thereby providing a competitive advantage of the product and also of the whole company.

Future work should consider the use of a pressure mapping system to identify the grasping strategies and study resulting contact forces and pressure distribution. Based on the findings, the shape of the handle could be altered to lower local peak contact pressure values below the critical level. The findings could be combined to propose smoothing function, which would allow development and manufacturing of a generalized tool handle for broad population with improved ergonomics.

## 4. Conclusions

Limitations within traditional design methods have led to the development of new user-centered design methods based on medical imaging which are presented in this paper. Presented quality and reliability of these methods can be used for the innovative design process to develop products which allow high consideration rate of biomechanical constraints and therefore provide best fit to the target population. The medical approach is especially suited for integrated CAD/FEA design process. With utilization of presented user-centered design methods based on medical imaging, it was possible to develop and manufacture an optimal sized and shaped tool handle for a target population with improved ergonomics.
